# Muscarinic receptor activation in colon cancer selectively augments pro-proliferative microRNA-21, microRNA-221 and microRNA-222 expression

**DOI:** 10.1371/journal.pone.0269618

**Published:** 2022-06-03

**Authors:** Shannon M. Larabee, Kunrong Cheng, Jean-Pierre Raufman, Shien Hu

**Affiliations:** 1 Department of Surgery, University of Maryland School of Medicine, Baltimore, Maryland, United States of America; 2 Department of Medicine, Division of Gastroenterology & Hepatology, University of Maryland School of Medicine, Baltimore, Maryland, United States of America; 3 VA Maryland Healthcare System, Baltimore, Maryland, United States of America; 4 Marlene and Stewart Greenebaum Cancer Center, University of Maryland School of Medicine, Baltimore, Maryland, United States of America; 5 Department of Biochemistry and Molecular Biology, University of Maryland School of Medicine, Baltimore, Maryland, United States of America; King Faisal Specialist Hospital and Research Center, SAUDI ARABIA

## Abstract

Overexpression of M3 subtype muscarinic receptors (M_3_R) hastens colon cancer progression. As microRNA (miRNA) expression is commonly dysregulated in cancer, we used microarrays to examine miRNA profiles in muscarinic receptor agonist-treated human colon cancer cells. We used quantitative RT-PCR (qPCR) to validate microarray results and examine miRNA expression in colon cancers and adjacent normal colon. These assays revealed that acetylcholine (ACh) treatment robustly induced miR-222 expression; miR-222 levels were three-fold higher in cancer compared to normal colon. In kinetic studies, ACh induced a 4.6-fold increase in pri-miR-222 levels within 1 h, while mature miR-222 increased gradually to 1.8-fold within 4 h. To identify post-M_3_R signaling mediating these actions, we used chemical inhibitors and agonists. ACh-induced increases in pri-miR-222 were attenuated by pre-incubating cells with atropine and inhibitors of protein kinase C (PKC) and p38 MAPK. Treatment with a PKC agonist, phorbol 12-myristate 13-acetate, increased pri-miR-222 levels, an effect blocked by PKC and p38 MAPK inhibitors, but not by atropine. Notably, treatment with ACh or transfection with miR-222 mimics increased cell proliferation; atropine blocked the effects of ACh but not miR-222. These findings identify a novel mechanism whereby post-M_3_R PKC/p38 MAPK signaling stimulates miR-222 expression and colon cancer cell proliferation.

## Introduction

Although colon cancer screening and other measures have reduced colorectal cancer incidence and mortality, data compiled by the American Cancer Society indicate that colon cancer continues to account for approximately 8 percent of U.S. cancer deaths; in 2020, 104,610 colon cancers were newly diagnosed, and 43,340 Americans died from advanced disease [1]. Moreover, it is worrisome that colon cancer incidence is rising in persons younger than 50 years [2]. While early endoscopic or surgical resection of primary cancers greatly improves prognosis, persons who succumb die primarily from advanced disease with unresectable metastases. Chemotherapeutic regimens, including immunotherapy, have limited efficacy and response durability [3].

Whereas the molecular events driving colon cancer progression from adenomas to carcinomas and metastasis remain obscure, emerging evidence suggests that in addition to serving as growth factors, muscarinic receptor agonists modulate the expression and activation of key molecules that promote cancer cell dissemination [4, 5]. Of the five muscarinic receptor subtypes, the M3 subtype (M_3_R) is a conditional oncogene when expressed in cells capable of proliferation [6]. M_3_R is widely expressed in the intestine and overexpressed in colon cancer [7, 8]. We previously showed that activation of M_3_R governs key hallmarks of colon cancer progression including cell proliferation, survival, migration, and invasion [7, 9–11]. In mouse models, M_3_R activation augments and M_3_R deficiency attenuates intestinal neoplasia [12–14]. Our previous work suggested that protein kinase C-α (PKC-α) and p38 MAPK are critical signal transduction nodes mediating the functional effects of M_3_R activation, including the induction of selected matrix metalloproteinases (MMPs) like MMP1, MMP7, and MMP10 that, by degrading extracellular matrix and other actions, promote cancer cell invasion [10, 15–17].

MicroRNAs (miRNAs) are ∼22 nucleotide, non-coding RNAs that play an important role in cell proliferation, apoptosis, and differentiation by regulating target gene expression [18]. They are critical regulators of intestinal homeostasis and their overexpression or dysregulation in colon cancer promotes disease progression [19]. Several oncogenic miRNAs, including miR-21 and miR-17-92 family members, target and inhibit the expression of endogenous tumor-suppressor genes that accelerate colon carcinogenesis [18–21]. Our previous work uncovered a novel mechanism whereby butyrate selectively suppresses miR-17-92, miR-221/222 and miR-106b clusters in colon cancer cells thereby permitting the expression of several cyclin-dependent kinase inhibitors, p21, p27 and p57 to modulate cell cycling [22, 23]. Collectively, these observations motivated us to test the hypothesis that post-M_3_R signaling regulates oncogenic miRNAs in colon cancer.

In the present study, we report that M_3_R activation selectively upregulates the expression of miR-21, miR-221, and miR-222 in human colon cancer cell lines. We also found these microRNAs were overexpressed in human colon cancer specimens. Mechanistic insights were gained using two human colon cancer cell lines; we uncovered a signaling pathway whereby muscarinic receptor agonists activate PKC which, in turn, induces p38 MAPK phosphorylation and augments levels of primary and mature miR-21, miR-221, and miR-222. Confirmatory evidence was obtained by showing miR-21, miR-221, and miR-222 induction was blocked by pre-incubating colon cancer cells with selective muscarinic, PKC, and p38 MAP kinase inhibitors. This regulatory mechanism suggests that M_3_R overexpression and activation in colon cancer contributes to miRNA dysregulation, e.g., upregulated oncogenic miR-21, miR-221, and miR-222, a novel post- M_3_R signaling action that is likely to play a role in mediating colon cancer progression.

## Materials and methods

### Human tissues

Tissue samples were obtained from individuals with colon cancer at the University of Chicago Medical Center under a protocol approved by the Institutional Review Board. Prior to tissue collection, informed consent was obtained from each subject. All clinical investigations using human subjects were conducted according to the principles expressed in the Declaration of Helsinki. At surgery, tissue was obtained from colon cancer and adjacent normal-appearing mucosa (more than 5 cm from the tumor border). These tissue samples were immediately rinsed in ice-cold phosphate-buffered saline (PBS) before cell lysis for RNA extraction.

### Cell lines and cell culture

HT-29 and H508, human colon cancer cell lines were authenticated and purchased from American Type Culture Collection (ATCC). H508 cells were grown in RPMI 1640 (Thermo Fisher Scientific) supplemented with 10% fetal bovine serum (FBS) plus antibiotics. HT-29 cells were grown in McCoy’s 5A medium (Thermo Fisher Scientific) supplemented with 10% FBS plus 50 μg/ml streptomycin and 50 U/ml penicillin. Cells were grown at 37°C, with 5% CO2 in a humidified incubator and passaged weekly at subconfluence after trypsinization. Prior to each experiment, the cells were incubated in serum-free medium without FBS for 24 hours for serum starvation.

### MicroRNA microarray

Total RNA was extracted from human cancer HT-29 cells or human colon samples using the mirVana miRNA Isolation Kit (Thermo Fisher Scientific) according to the manufacturer’s protocol. Human miRNA profiles were analyzed using Exiqon miRNA array 7th Gen (Product #208500) for HT-29 cells and mirVana miRNA Bioarrays v. 2 (Thermo Fisher Scientific) for human colon samples according to the manufacturer’s instructions.

### Reagents and antibodies

Antibodies for total p38, phospho-p38, and β-actin were purchased from Cell Signaling. Chemicals were purchased from Sigma-Aldrich as follows: acetylcholine (ACh), atropine, phorbol 12-myristate 13-acetate (PMA), PKC inhibitor Gö 6976 and p38 MAP kinase inhibitor SB203580.

### Immunoblot analysis

After the indicated treatments, cells were lysed in a solution containing 20 mM Tris-HCl, 100 mM NaCl, 5 mM MgCl2, 1 mM phenylmethylsulfonyl fluoride, 1 mM NaF, 1 mM NaVO3, 1 mM EDTA, 1% Triton X-100, 1 μg/ml pepstatin, and 1 μg/ml leupeptin. Cell lysates were centrifuged at 15,000 × g at 4°C for 10 min. Supernatants were collected and protein concentration was determined by the BCA method (Thermo Fisher Scientific). Proteins were separated by SDS-PAGE and transferred to nitrocellulose membranes that were probed against total (Cell Signaling catalog #9212), phospho-p38 for sites p-T180 and p-Y182 (Cell Signaling catalog #9211). To confirm equal protein loading, blots were stripped and re-probed with antibody to β-actin. Image quantification was performed by scanning densitometry using NIH Image J 1.50i software. In most experiments, conditions were normalized and expressed relative to the positive control.

### Quantitative real-time PCR (qPCR)

Total RNA was extracted from pelleted cells by TRIzol (Thermo Fisher Scientific) according to the manufacturer’s instructions. Universal complementary DNA was synthesized from total RNA samples using the NCode Vilo miRNA cDNA Synthesis Kit (Invitrogen) or TaqMan Advanced miRNA cDNA Synthesis Kit (Thermo Fisher Scientific). Real-time PCR was performed with ABI StepOnePlus real-time PCR system (Applied Biosystems). The two-step quantification cycling protocol (2 min at 50°C, 10 min at 95°C and then 40 cycles of 95°C for 15 s and 60°C for 60 s) was used. PCR specificity was confirmed by melting curve analysis. For miRNA, TaqMan Advanced miRNA Assays specific for miR-21, miR-221 and miR-222 were used according to the manufacturer’s protocol. All miRNAs were normalized to a small nucleolar RNA, *RNU48*. For primary miRNA and GAPDH mRNA, Veriquest Sybr Green qPCR Master (Affymetrix) with specific primers were used. All pri-miRNAs were normalized to GAPDH. Sense and antisense PCR primers for qPCR were as follows: *pri-miR-21*: 5’-TCTTTCATCTGACCATCCATATCC-3’ and 5’- CAGACAGAAGGACCAGAGTTTC-3’; *pri-miR-221/222*: 5’-ACACACTCACACACACACTC-3’ and 5’-CCCTAGAACTTGACTCTCTCCT-3’; *pri-miR-25*: 5’-GACACCCTTGTTCTGGCTTTA-3’ and 5’-ATTGCACTTGTCTCGGTCTG; *pri-miR-17-92a*: 5’-AGTGAAGGCACTTGTAGCATTA-3’ and 5’-GCACTAGATGCACCTTAGAACA-3’; *GAPDH*, 5-CTCCTCACAGTTGCCATGTA-3′ and 5′-GTTGAGCACAGGGTACTTTATTG-3′. For quantification, the fold-change of mature or primary miRNA in experimental relative to control samples was determined by the 2^-ΔΔCt^ method.

### Cell proliferation assay

Cell proliferation was measured using the WST-1 Cell Proliferation Assay Kit (Takara) according to the manufacturer’s instructions. HT-29 cells were cultured on a 96-well flat-bottom plates. After reaching 50% confluence, cells were treated with ACh or miR-222 mimic transfections for 24 h with or without pretreatment with atropine. Plates were read on a microplate reader at 450 nm before and 45 min after adding the WST-1 reagent. The reference wavelength was 650 nm. Cell proliferation rates were calculated according to the manufacturer’s protocol.

### Cell transfection with miRNA

Lipofectamine 3000 (Thermo Fisher Scientific) transfection reagent was used to transfect HT-29 cells with miR-222 mimics (*mirVana* miRNA mimic, Thermo Fisher Scientific) according to the manufacturer’s protocol. Cells were transfected for 24 h prior to further experiments.

### Statistical analysis

Data are presented as mean ± SE of at least three separate experiments and analyzed by two-tailed unpaired Student’s *t*-test. *P* < 0.05 was considered statistically significant.

## Results

**Fig 1:**
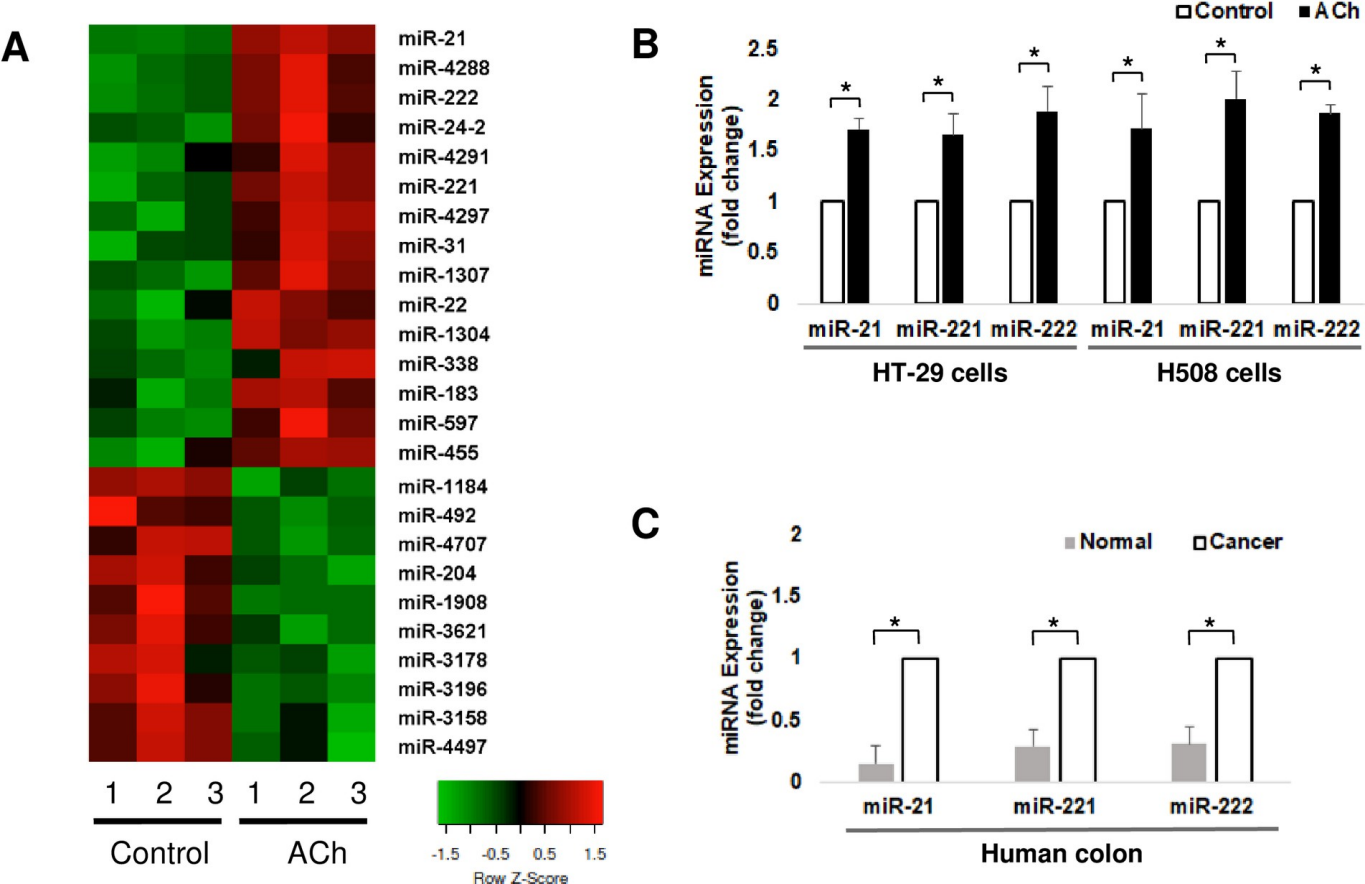
Treatment with a muscarinic receptor agonist, acetylcholine (ACh), alters the expression of microRNAs (miRs) in human colon cancer cells. **A)** miRNA microarray profiles were performed on RNA extracted from HT-29 cells treated with vehicle or 100 μM ACh for 4 h. Data were normalized using the global Lowess regression algorithm and are expressed as log base 2 transformed ratios of the sample signal to the control reference pool signal. miRNAs with statistically significant changes (p<0.05) after ACh treatment are shown in the heat map; red represents increased expression compared to the pooled reference control, and green represents decreased expression. **B)** Changes in miR-21, miR-221, and miR-222 expression were confirmed by qPCR in HT-29 and H508 human colon cancer cells. Results are means ± SE, n  =  4. *, indicates p<0.05 for the test sample compared to control. **C)** Expression of miR-21, miR-221, and miR-222 in sporadic colonic cancers and adjacent normal-appearing tissue were measured by microarray. Results are means ± SE, n  =  4. *, indicates p<0.05 for expression in cancer compared to normal.

In our initial experiments, we used well-characterized HT-29 human colon cancer cells that abundantly express M_3_R and respond robustly to muscarinic agonist treatment [16, 24]. To explore the effects of activating M_3_R on miRNA expression levels in this colon cancer cell line, after treating cells with acetylcholine (ACh), miRNA expression profiles were measured using a microarray. Vehicle-treated HT-29 cells served as a control. After ACh treatment for 4 h, the resulting heatmap revealed increased expression of 15 miRNAs and decreased expression of 10 miRNAs (Fig 1A). In terms of ACh-induced expression, miR21 and both members of the miR-221/222 cluster were among the miRNAs most substantially increased. miR-21, miR-221, and miR-222 are oncogenic miRNAs previously shown to promote colon cancer progression [19, 25, 26].

Expression levels of miR-21, miR-222, and miR-221 were confirmed in the same samples using qPCR (Fig 1B). Using qPCR, we also observed similar findings in H508 cells, another well-characterized human colon cancer cell line with high expression levels of M_3_R [16, 24]. As shown in Fig 1B, in H508 cells ACh treatment robustly up-regulated expression of miR-21, miR-221, and miR-222. Next, using a microarray, we assessed expression levels of miR-21, miR-221, and miR-222 in sporadic human colon cancers using adjacent normal colon as a comparator (Fig 1C). Again, the expression levels of oncogenic miR-21, miR-221, and miR-222 were dramatically increased in cancer compared to the normal controls, a finding consistent with previous reports of miRNA expression in colon cancer [19, 27]. From this set of experiments, we concluded that M_3_R activation in colon cancer cells further increases the levels of oncogenic miRNAs that already have high basal expression levels. These findings support the conclusion that in colon cancer M_3_R overexpression and activation contribute to dysregulated miRNA expression. Moreover, these effects on oncogenic miRNA expression are likely to contribute to the adverse effects of M_3_R activation on colon cancer progression.

**Fig 2:**
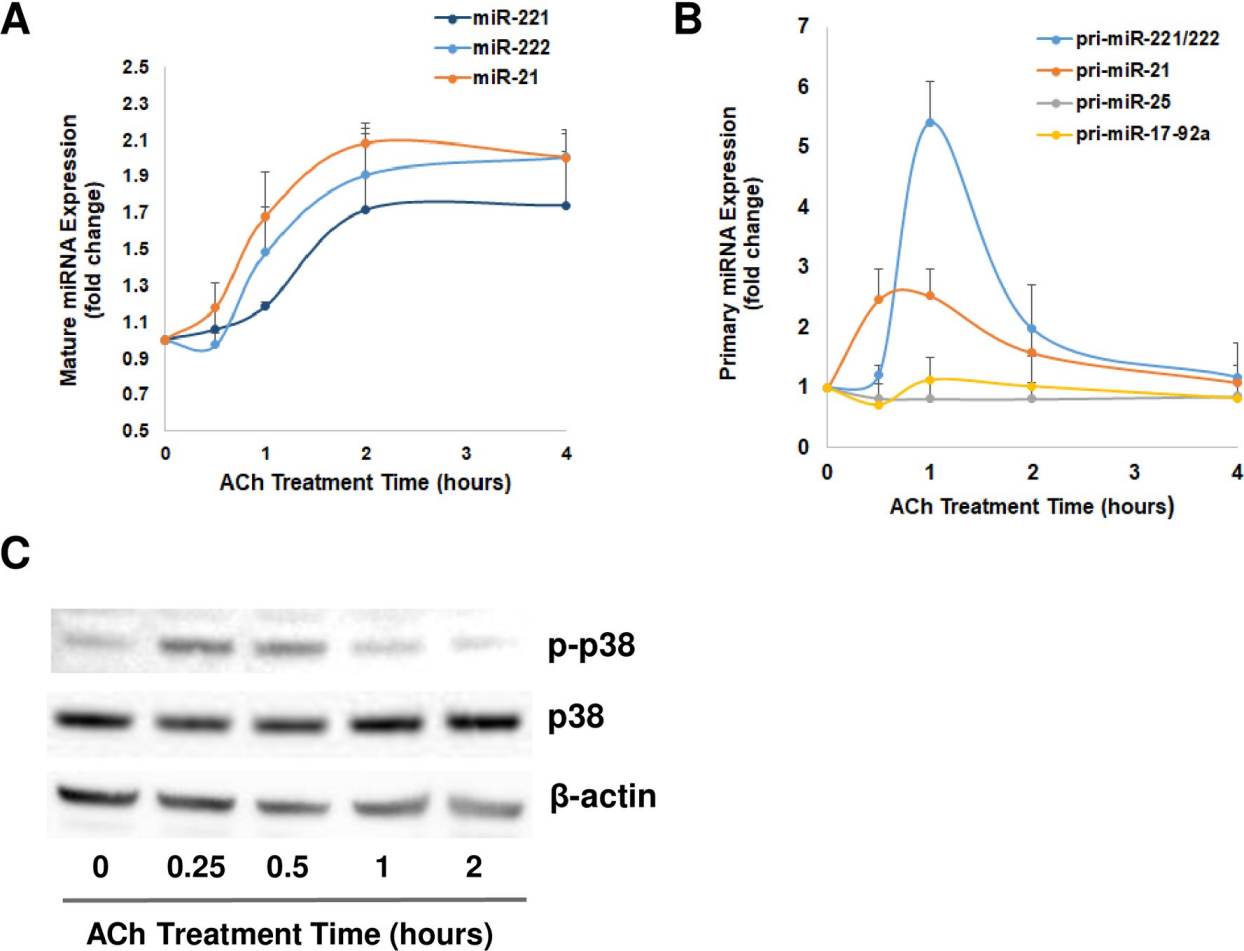
Time course of changes in miRNA, primary miRNA and p38 phosphorylation levels following treatment with ACh. HT-29 human colon cancer cells were treated with 100 μM ACh for up to 4 h. Cells were harvested for analysis at 0.5, 1, 2, and 4 h after treatment. The abundance of **A)** mature miR-21, miR-221, and miR-222 and **B)** pri-miR-21, pri-miR-221/222, pri-miR-25, and pri-miR-17-92a were measured by qPCR. Bars represent means ± SEM. *n*  =  3. **C)** Protein levels of phosphorylated p38 MAP kinase (p-p38), total p38, and β-actin were analyzed by immunoblotting. The image shown is representative of three individual experiments.

To explore the regulatory mechanisms of post-M_3_R activation on miRNAs, we examined the effects of treating HT-29 cells with ACh for four hours on the expression of mature miRNAs and their initial transcripts, primary miRNAs. miRNA levels were measured 0, 0.5, 1, 2, and 4 h after ACh treatment. The levels of mature miR-21, miR-221, and miR-222 increased gradually 30 min after treatment and after two hours plateaued at 1.6- to 2.0-fold increases compared to baseline (Fig 2A). As shown in Fig 2B, we observed a rapid increase in pri-miR21 within one hour after ACh treatment that peaked at 2.5-fold over basal levels. miR-221 and miR-222 originate from the same primary miRNA, pri-miR-221/222. Pri-miR-221/222 showed a steep increase within one hour and peaked over 5-fold above basal levels one hour after treatment. At four hours after treatment, both pri-miR-21 and pri-miR-221/222 levels had declined close to baseline. Levels of pri-miR-25 and pri-miR17-92a, measured as controls, were not altered by ACh treatment (Fig 2B). This result again supports the specificity of ACh-induced changes on microRNA expression levels.

Next, we explored post-M_3_R signaling pathways that mediate changes in microRNA expression. The PKC-p38 MAPK pathway is a major post-M_3_R signal transduction mechanism which activates transcription of various downstream genes [16, 17]. We hypothesized that this pathway was likely to mediate post-M_3_R activation induced changes in the expression of miRNA genes. To test this hypothesis, we analyzed p38 MAPK phosphorylation (p-p38 MAPK) levels after ACh treatment. As shown in Fig 2C, after ACh treatment the levels of p38 MAPK phosphorylation increased within 15 min; in contrast, levels of total p38 MAPK were not altered. The chronological sequence of p38 MAPK phosphorylation and increases in primary and mature miRNA after ACh treatment supports the likelihood that in human colon cancer cells phosphorylation (activation) of p38 MAPK downstream of M_3_R activation induces transcription of miR-21, miR-221 and miR-222.

**Fig 3:**
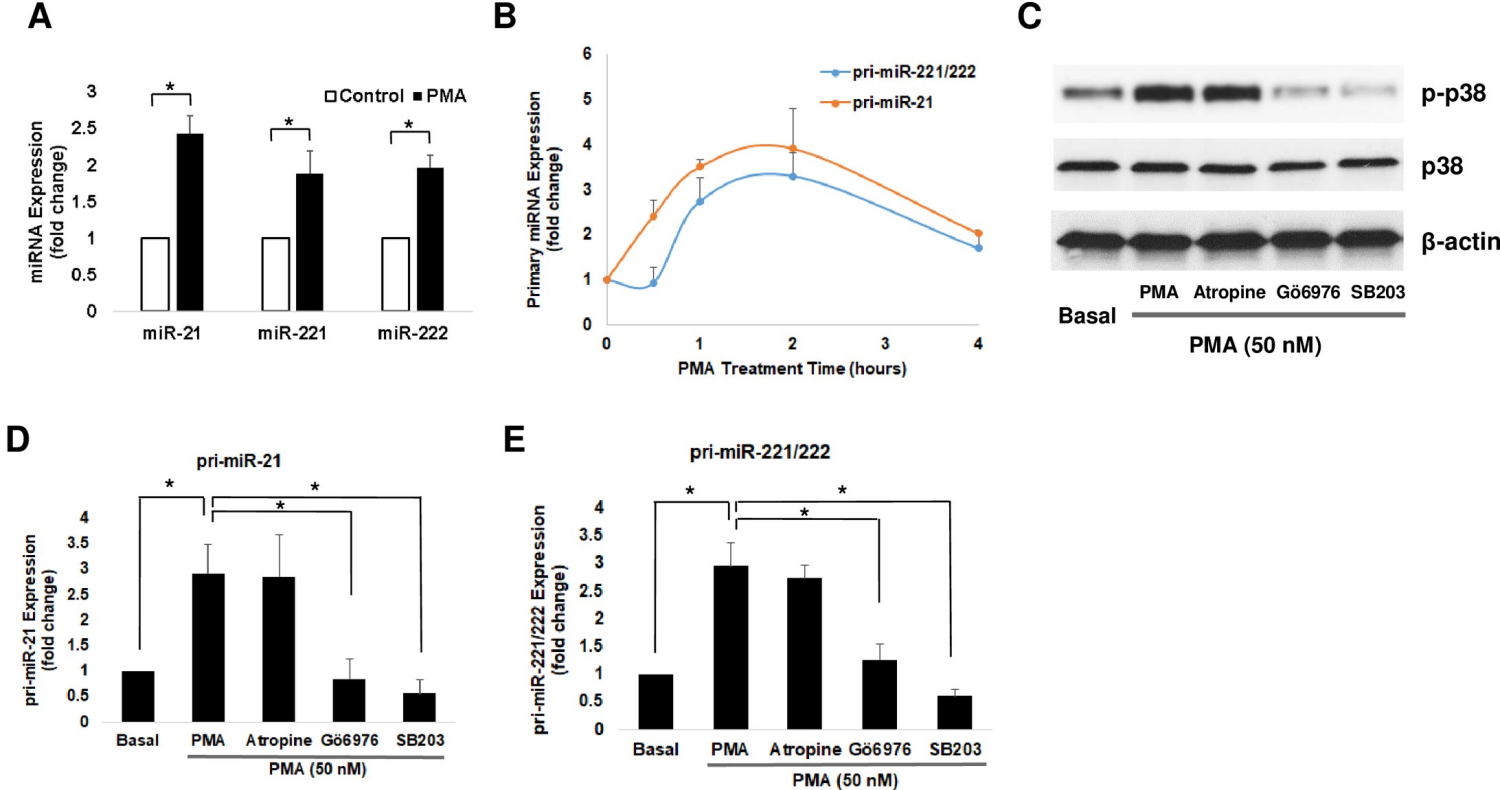
Activating protein kinase C (PKC) up-regulates mature and primary miR-21, miR-221 and miR-222 in human colon cancer cells. HT-29 cells were treated with 50 nM PMA for up to 4 h. **A)** The abundance of mature miR-21, miR-221 and miR-222 was measured by qPCR 4 h after treatment. Bars represent means ± SEM. *n*  =  4. *, indicates p<0.05 for test samples compared to control. **B)** Cells were harvested for analysis at 0.5, 1, 2, and 4 h after treatment. The abundance of pri-miR-21 and pri-miR-221/222 was measured by qPCR. Bars represent means ± SEM. *n*  =  3. **C)** Cells were pre-incubated for 45 min with 5 μM atropine to inhibit M_3_R activation, 5 μM Gӧ6976 to inhibit PKC activation, and 10 μM SB203580 (SB203) to inhibit p38 activation before adding 50 mM PMA. After 15-min incubation with PMA, protein levels of phosphorylated p38 MAP kinase (p-p38), total p38, and β-actin were analyzed by immunoblotting. The image shown is representative of three individual experiments. **D)** Pri-miR-21 and **E)** pri-miR-221/222 levels were measured by qPCR in HT-29 cells with inhibitor pretreatment prior to 60-min incubation of PMA. Bars represent means ± SEM. *n*  =  4 *, *P* < 0.05 compared to 50 mM PMA alone.

To investigate further the role of PKC-p38 MAPK activation in mediating post-M_3_R induction of miRNAs, we incubated HT-29 cells with phorbol 12-myristate 13-acetate (PMA) to activate PKC directly. As shown in Fig 3A, PMA treatment for 4 h significantly increased mature miR-21, miR-221, and miR-222 by 1.8- to 2.4-fold compared to control. Within 2 h, PMA induced a 3- to 4-fold increase in pri-miR-21 and pri-miR-221/222 levels (Fig 3B). A short treatment of PMA for 15 min robustly induced p38 MAPK phosphorylation without altering total p38 MAPK levels (Fig 3C). Using previously validated selective chemical inhibitors, we confirmed the effect of PMA in selectively activating PKC. Inhibition of M_3_R activation by atropine upstream to PKC had no effects on PMA-induced p38 phosphorylation. In contrast, inhibition of PKC activation by Gӧ6976 or inhibition of p38 MAPK activation by SB203580 completely blocked PMA-induced p38 phosphorylation (Fig 3C). PMA-induced activation of PKC recapitulated the effects of ACh treatment on both primary and mature miR-21, miR-221 and miR-222, providing further evidence to support the role of the M_3_R-PKC-p38 MAPK pathway in the regulation of these microRNA levels.

**Fig 4:**
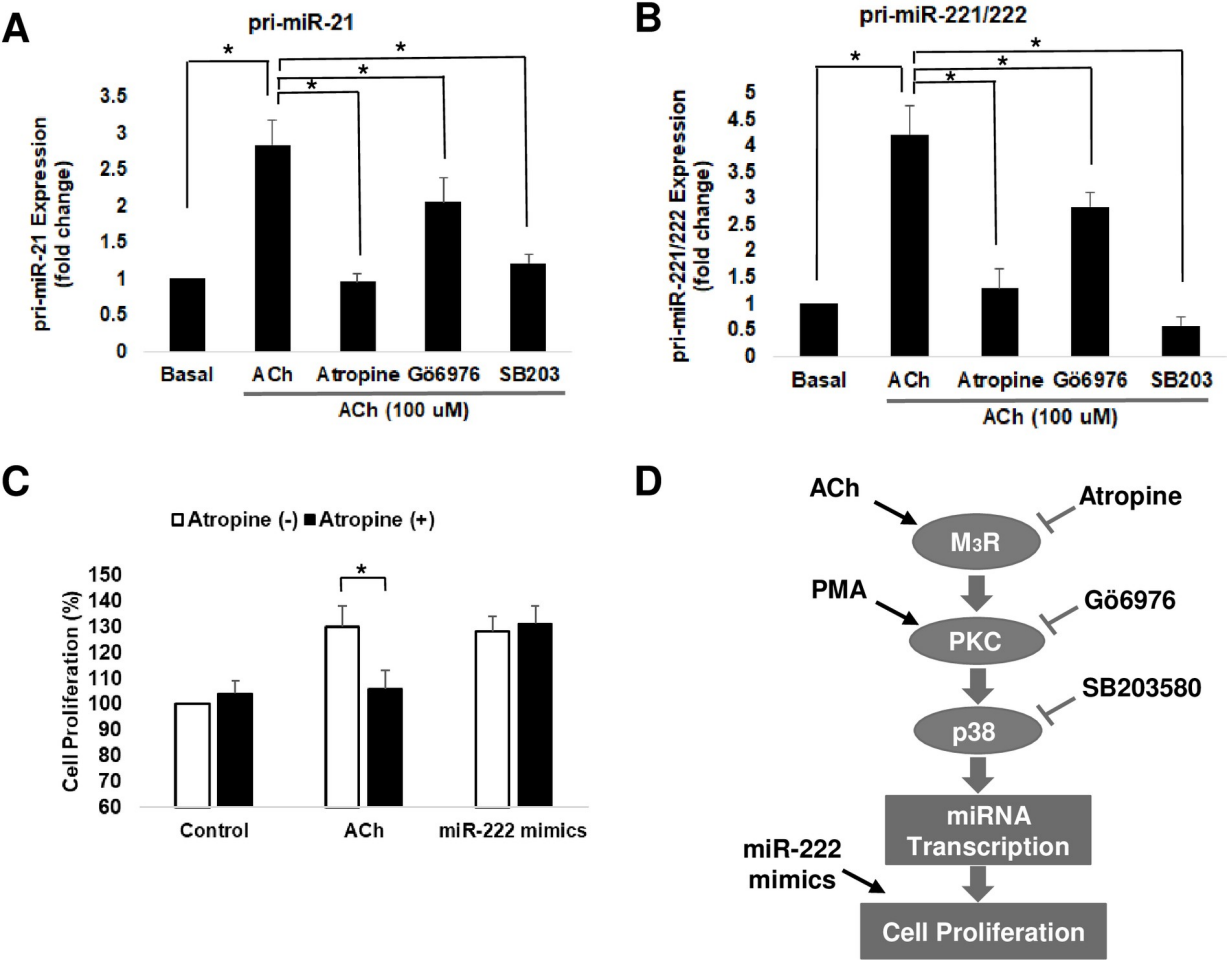
ACh-induced upregulation of miRNA expression and cell proliferation is mediated by activation of PKC and p38. **A)** Pri-miR-21 and **B)** pri-miR-221/222 levels were measured by qPCR in HT-29 cells. Cells were pre-incubated for 45 min with 5 μM atropine to inhibit M_3_R activation, 5 μM Gӧ6976 to inhibit PKC activation, and 10 μM SB203580 (SB203) to inhibit p38 activation. Then, 100 μM ACh was added for an additional 60-min incubation. Bars represent means ± SEM. *n*  =  4. *, indicates p< 0.05 compared to 100 μM ACh alone. **C)** HT-29 cells were pre-incubated for 45 min with 5 μM atropine before adding 100 μM ACh or transfecting cells with miR-222 mimics for an additional 24 h. Cell proliferation was measured using the WST-1 assay. Bars represent means ± SEM. *n*  =  4 *, *P* < 0.05 compared to cells without atropine. **D)** Working model for post-M_3_R signal transduction mediating muscarinic receptor agonist-induced increases in selected miRNAs in colon cancer. The agonists, inhibitors, and miRNA mimics used to test each step in the proposed signaling pathway are shown.

Next, we used the same selective chemical inhibitors to uncover the intermediary signaling molecules that regulate miRNA levels in colon cancer cells. As shown in Fig 3D and 3E, Gӧ6976 and SB203580, but not atropine, strongly attenuated PMA-induced upregulation of both pri-miR-21 and pri-miR-221/222, consistent with the effects on p38 phosphorylation (Fig 3C). In contrast, all three inhibitors—atropine, Gӧ6976, and SB203580—inhibited the effects of ACh on these primary miRNAs (Fig 4A and 4B).

Lastly, to confirm that microRNAs induced by muscarinic agonists can alter cell function, we measured their effects on colon cancer cell proliferation. Using the WST-1 cell proliferation assay, we compared the effects of transfecting cells with exogenous miR-222 mimics to those of ACh, with or without pretreatment with atropine (Fig 4C). Notably, miR-221 and miR-222 have identical seed sequences and share the same target proteins and associated cell functions. As expected, ACh treatment induced a statistically significant increase in HT-29 cell proliferation that was blocked by pre-treating cells with atropine (Fig 4C). In contrast, whereas transfecting cells to overexpress miR-222 mimics also increased HT-29 cell proliferation rate, with similar efficacy as ACh, this effect was not altered by pre-treating cells with atropine (Fig 4C).

Collectively, as summarized in the working model shown in Fig 4D, our findings using highly selective agonists and inhibitors provide evidence that activating M_3_R, PKC, or p38 MAPK increases the levels of selected microRNAs—miR-21, miR-221, and miR-222—in human colon cancer cells. Moreover, as shown by our experiments using miR-222 mimics, these changes in microRNA levels are sufficient to augment colon cancer cell proliferation.

## Discussion

Of the five muscarinic receptor subtypes, M_3_R are commonly overexpressed in colon cancer [7]. Like the other muscarinic receptor subtypes, M_3_R can be activated by the traditional muscarinic receptor ligand, ACh, synthesized and released by neurons, but also by non-neuronal tissues including colon cancer cells [28] and immunocytes [29]. Moreover, other endogenous molecules, such as selected bile acids, can modulate the activity of muscarinic receptors [30], perhaps by allosteric mechanisms [31]. Post-M_3_R signal transduction involving complex, interacting pathways ultimately induce the transcription of genes that modulate colon cancer cell proliferation, survival, migration, invasion, and metastasis [17, 32]–all key hallmarks of cancer progression [33].

Dysregulated miRNA expression is also frequently detected in colon cancer, but the underlying mechanisms are not well understood and, to our knowledge, a relationship with MR signaling has not previously been reported. Hence, we were intrigued by our finding that miR-21, miR-221, and miR-222 were among the miRNAs most prominently increased in a microarray assay performed after treating colon cancer cells that overexpress M_3_R with muscarinic agonists (Fig 1). Notably, miR-21, miR-221, and miR-222 are oncogenic miRNAs whose upregulation in colon cancer is associated with increased metastatic potential and poor clinical outcomes [19, 27]. Pursuing these observations allowed us to contribute novel insights into the mechanisms whereby post-M_3_R activation stimulates the progression of colon cancer [4, 5]. The novel finding that non-coding miRNA levels are upregulated in colon cancer following treatment with ACh extends our understanding of post-M_3_R signal transduction mechanisms mediating colon cancer progression and provides novel therapeutic targets.

The PKCα-p38 MAPK axis is a major signaling pathway downstream of M_3_R [16, 17]. Previously, we identified a key role for PKCα-p38 MAPK signaling in post-M_3_R induction of matrix metalloproteinase-1 (*MMP1*) gene expression [16]. In the present study, we found that both ACh and a direct activator of PKCα, PMA, induced p38 MAPK activation (phosphorylation) and increased expression levels of primary and mature miR-21 and miR-221/222 (Figs 2 and 3). Confirmatory experiments showed that preincubating cells with inhibitors of M_3_R, PKCα, and p38 MAPK, blocked these effects. Control experiments showed that whereas preincubating cells with atropine blocked the effects of ACh, this did not alter the effects of directly activating PKCα. Collectively, these internally consistent results substantiate the critical role of the M_3_R-PKC-p38 MAPK axis in modulating miR-21 and miR-221/222 expression in colon cancer. These findings substantiate the potential therapeutic promise of attenuating M_3_R activation and post-M_3_R signaling in colon cancer [31].

Post-ACh treatment time-course experiments (Fig 2) revealed nearly immediate p38 phosphorylation and increases in primary miRNA, followed by increases in mature miRNA, suggest transcriptional regulation of miRNA expression. In future studies beyond the scope of the current investigation, we plan to identify transcriptional factors downstream of the M_3_R-PKCα-p38 MAPK axis and test their effects on miRNA promoter activities using reporter assays. cMyc, a well-characterized colon cancer transcription factor, induces miR-17-92a expression. Previously, we found butyrate down-regulates miR-17-92a transcription in colon cancer by diminishing cMyc [22], but we did not detect post-ACh treatment changes in miR-17-92a levels (Fig 2B). Thus, the role of oncogenic transcription factors in miRNA dysregulation in colon cancer warrants further investigation.

As regulators of gene expression selectively increased by ACh treatment, miR-21 and miR-221/222, are likely to mediate multiple post-M_3_R effects that favor colon cancer progression. miR-21, a prominent oncomiR in colon cancer, demonstrates pro-tumorigenic properties in many solid cancers [19, 34]. Several validated miR-21 target genes are tumor suppressors, e.g., programmed death protein 4 (PDCD4) [34, 35]. miR-221 and miR-222 overexpression promotes cell proliferation by targeting key cell cycle regulators, e.g., cyclin-dependent kinase inhibitors p27 and p57 [36, 37]. Among the many established and predicted protein targets of miR-21 and miR-221/222, screening common target proteins of these miRNAs that interact with known post-M_3_R downstream factors will be the focus of our next phase of study. Examples include reversion-inducing-cysteine-rich protein (RECK) and metalloproteinase inhibitor 3 (TIMP3), which are post-transcriptionally regulated by both miR-21 and miR-221/222 [26, 37–39]. Emerging evidence also suggests that miR-21, miR-221, and miR-222 are immunomodulators [40]. As inflammatory signaling is a key driver of colon cancer, particularly regarding colitis-associated cancer [40], this observation suggests that it would be worthwhile to explore the role of the M_3_R-PKC-p38 MAPK axis in inflammation-associated colon cancer.

## Conclusions

Our findings reveal that M_3_R signaling via the PKC-p38 MAPK pathway selectively induces expression of oncogenic miR-21, miR-221 and miR-222 in colon cancer cells, a mechanism contributing to miRNA dysregulation in colon cancer. These oncogenic miRNAs are likely to mediate some post-M_3_R effects on colon cancer progression. Thus, this novel regulatory mechanism provides new insights into colon cancer progression and identifies potential therapeutic targets. Studies to discover common protein targets and additional colon cancer cell functions modulated by these miRNAs will follow. For example, investigations of the interaction of these microRNAs with the genes for M_3_R-activated MMPs [15] will fill additional gaps in knowledge and allow us to harness the therapeutic potential of targeting these post-M_3_R mechanisms to treat colon cancer.

## Supporting information

S1 File Original uncropped and unadjusted images for Figs 2C and 3C are shown. Red boxes identify immunoblots shown in the manuscript.
(PDF)

## Abbreviations

ACh: acetylcholine
miR: microRNA
M_3_R: M3 subtype muscarinic receptor
PKC: protein kinase C
MAPK: mitogen-activated protein kinase
PMA: 12-myristate 13-acetate
