# Supplementary figures and images for: Muscarinic receptor activation in colon cancer selectively augments pro-proliferative microRNA-21, microRNA-221 and microRNA-222 expression
Source: PLoS One. 2022 Jun 3;17(6):e0269618. doi: 10.1371/journal.pone.0269618 (PMC9165902; doi:10.1371/journal.pone.0269618)

Figure 2C

Molecular  
Weight (k Da)

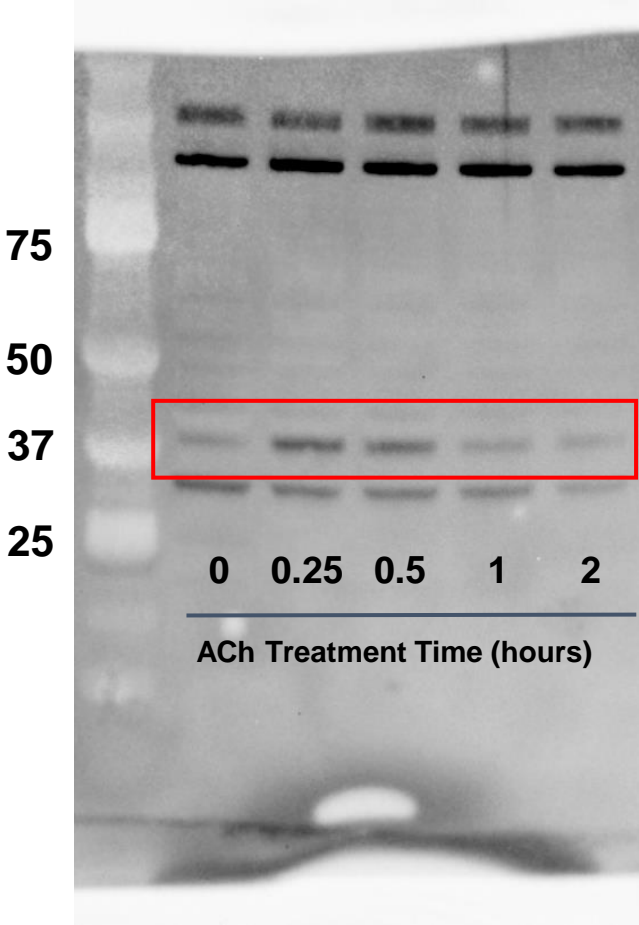

← phospho p38

Figure 2C

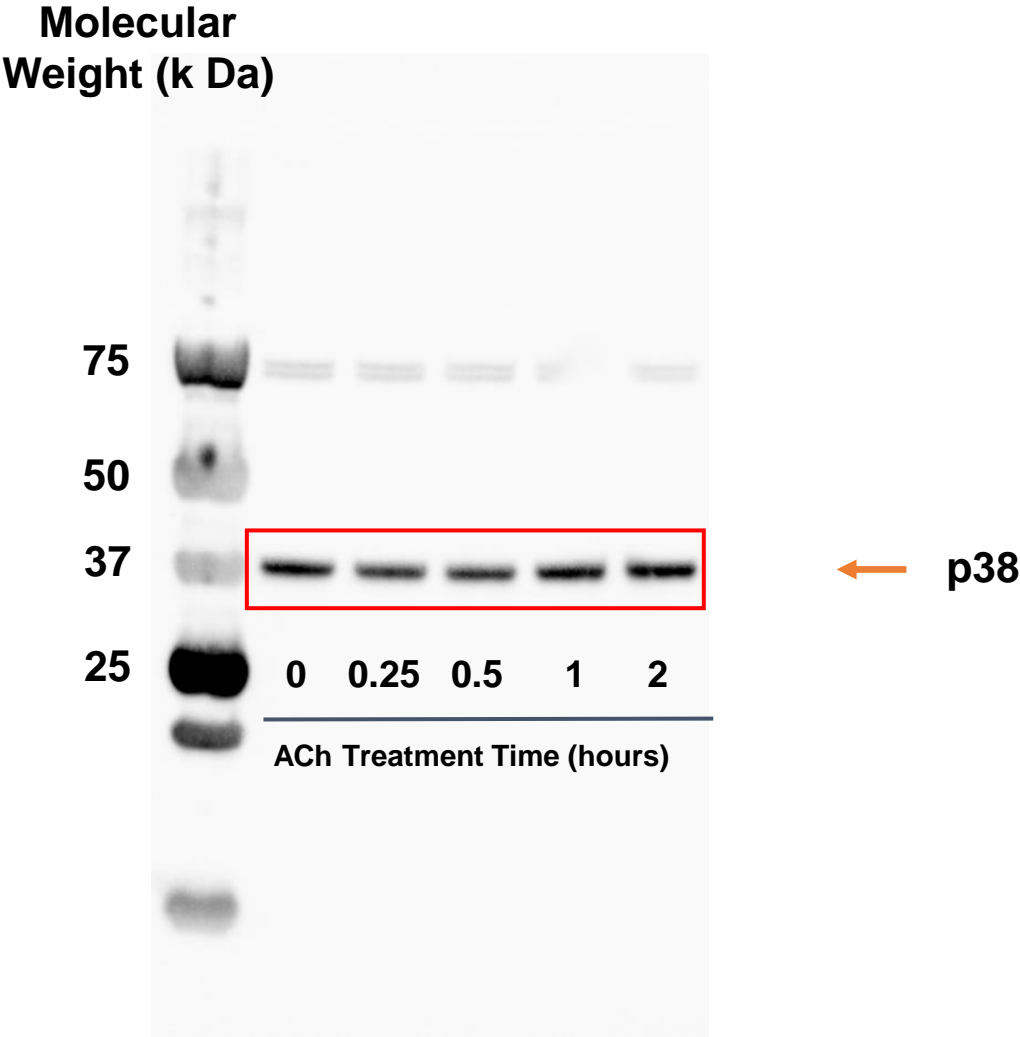

Figure 2C

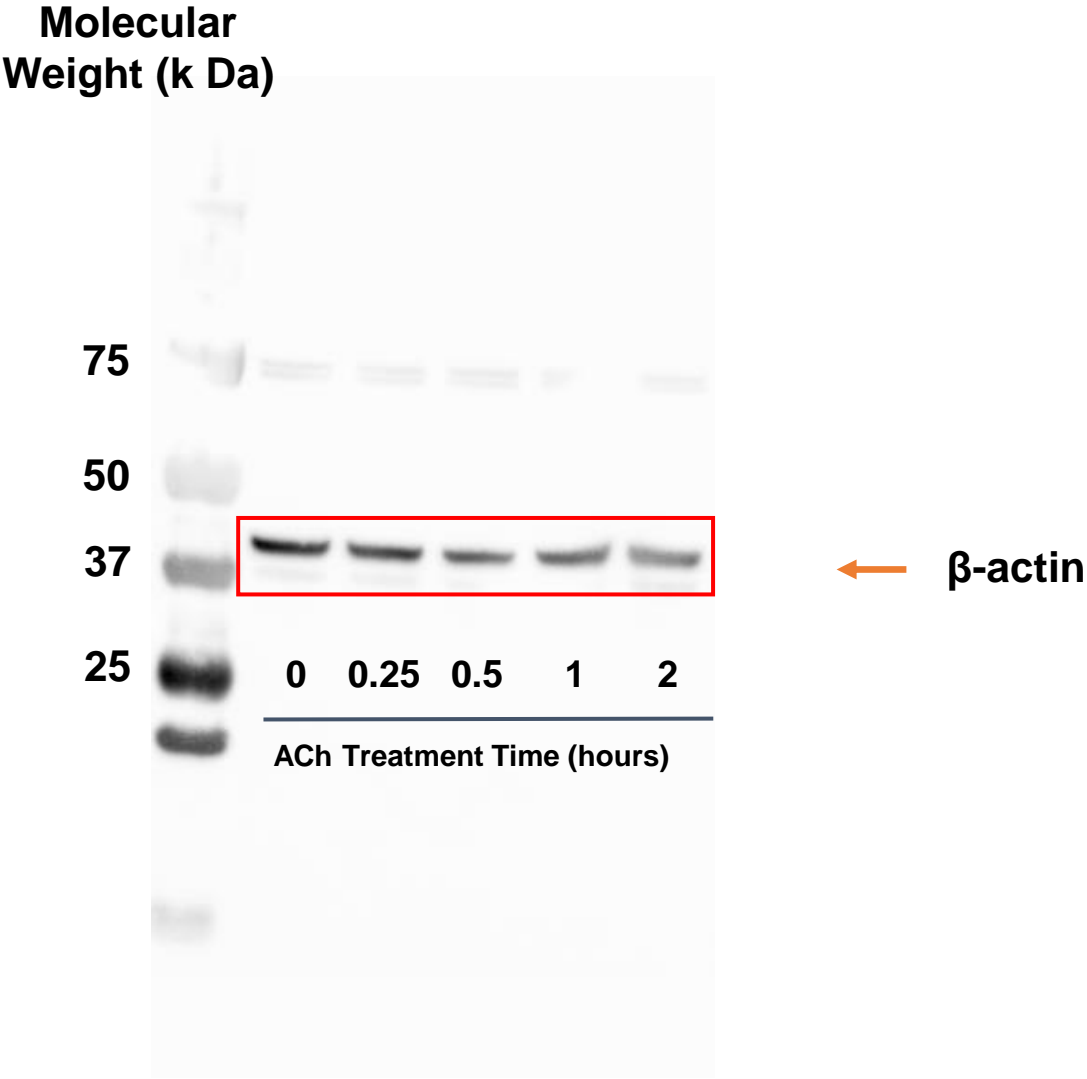

Figure 3C

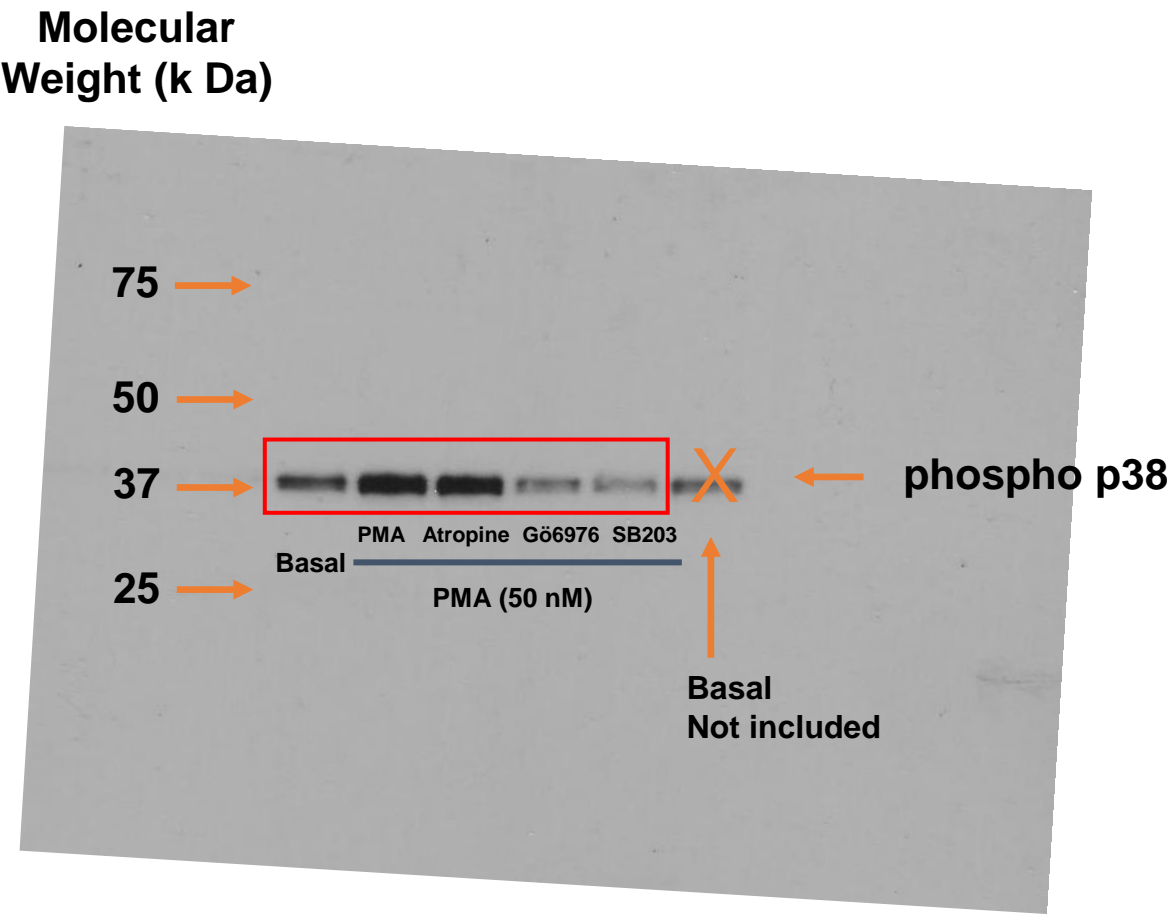

Figure 3C

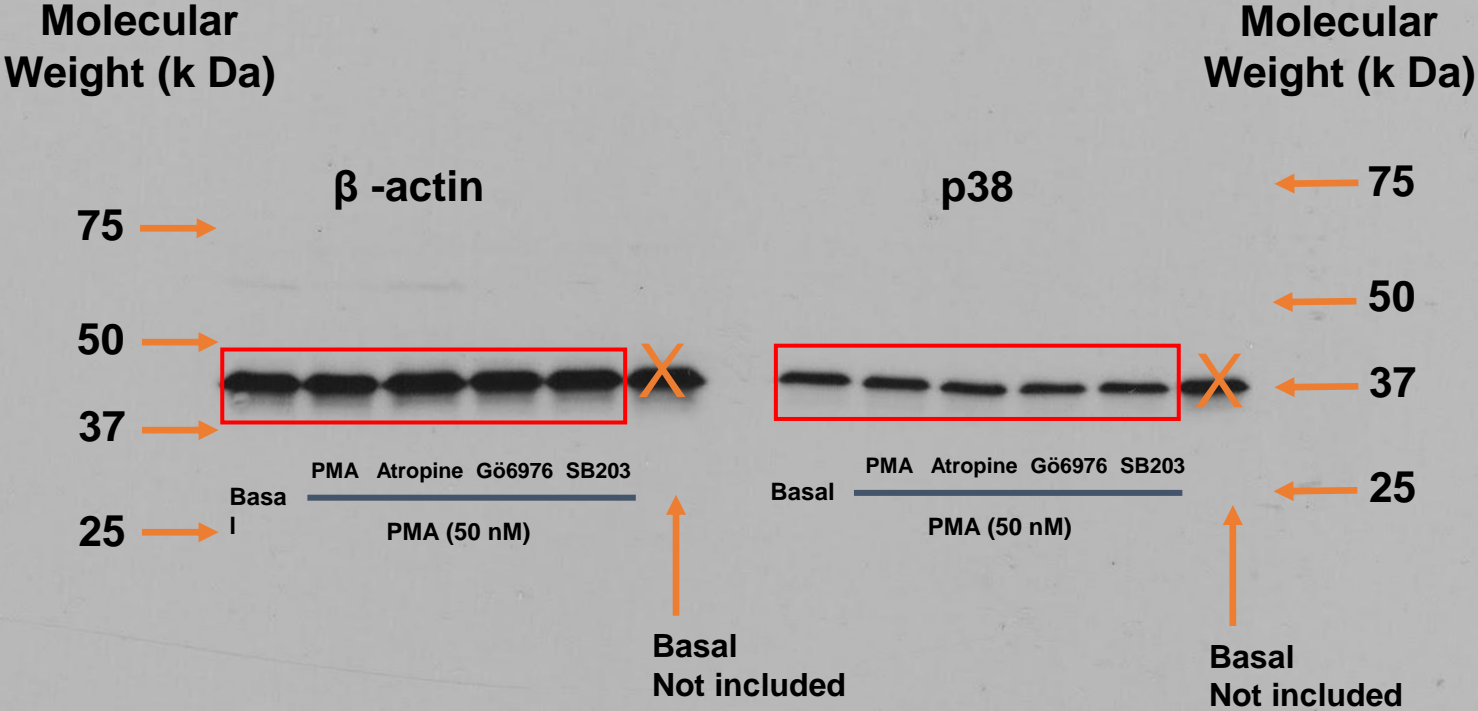

Supplement: S1 File — Red boxes identify immunoblots shown in the manuscript. (PDF) [file pone.0269618.s001.pdf]
